# Idiosyncratic development of sensory structures in brains of diapausing butterfly pupae: implications for information processing

**DOI:** 10.1098/rspb.2017.0897

**Published:** 2017-07-05

**Authors:** Philipp Lehmann, Sören Nylin, Karl Gotthard, Mikael A. Carlsson

**Affiliations:** 1 Department of Zoology, Stockholm University, SE-106 91 Stockholm, Sweden; 2 Bolin Centre for Climate Research, Stockholm University, SE-106 91 Stockholm, Sweden

**Keywords:** energetics, information processing, neurobiology, sensory system, phenology

## Abstract

Diapause is an important escape mechanism from seasonal stress in many insects. A certain minimum amount of time in diapause is generally needed in order for it to terminate. The mechanisms of time-keeping in diapause are poorly understood, but it can be hypothesized that a well-developed neural system is required. However, because neural tissue is metabolically costly to maintain, there might exist conflicting selective pressures on overall brain development during diapause, on the one hand to save energy and on the other hand to provide reliable information processing during diapause. We performed the first ever investigation of neural development during diapause and non-diapause (direct) development in pupae of the butterfly *Pieris napi* from a population whose diapause duration is known. The brain grew in size similarly in pupae of both pathways up to 3 days after pupation, when development in the diapause brain was arrested. While development in the brain of direct pupae continued steadily after this point, no further development occurred during diapause until temperatures increased far after diapause termination. Interestingly, sensory structures related to vision were remarkably well developed in pupae from both pathways, in contrast with neuropils related to olfaction, which only developed in direct pupae. The results suggest that a well-developed visual system might be important for normal diapause development.

## 1. Background

Different dormancy stages are common escape mechanisms in animals living in seasonal environments that are characterized by predictable temporal variability in levels of abiotic and biotic stress [1]. In insects, diapause is a particularly deep form of dormancy that occurs at a species-specific developmental stage (egg, larva, pupa or adult). Diapause may further be obligatory or facultative, the latter meaning that individuals make a choice to either develop directly without diapause or to undergo diapause development [1], thus forgoing a reproductive opportunity. In species having several annual generations, there are often a number of non-diapausing, directly developing generations (generally 1–2), followed by a final generation that initiates winter diapause [1].

Diapause itself is a dynamic developmental trajectory with several phases [2]. After diapause is induced, two main phases ensue: the first is diapause maintenance, during which insects are non-responsive to external conditions. At some point during diapause development, usually during mid-winter, the maintenance state is terminated and shifts to the second phase, post-diapause quiescence, during which insects again become responsive to external conditions. Still, after termination, the diapause phenotype is typically maintained by cold winter conditions, and consequently, diapause shifts from being internally to externally maintained [3]. In many insects, chilling is needed for diapause maintenance to progress to termination, and the timing of termination shows adaptive variation across latitudes [4,5]. Unlike diapause induction, which has been studied intensely on several functional levels [1], the mechanisms of diapause time-keeping and termination are poorly understood [3,6,7]. In studies on biological rhythms and timing mechanisms, it is often suggested that the circadian clock and a functional neural network are needed for normal time-keeping [8]. However, the development of insect brains during diapause has never been studied.

While brains of insects that overwinter in the adult stage develop prior to winter, and thus likely have fully developed sensory and time-keeping capacities [9], it is unclear if the same applies for insects that overwinter as pupae. During metamorphosis, the insect brain undergoes a dramatic transformation [10]. Many larval neurons persist during metamorphosis until adulthood in insects, though often with rewiring of neural processes [11]. However, most neurons associated with sensory systems are developed de novo from embryonic neuroblasts during pupation [11]. This is particularly true for the visual and olfactory systems, as both the compound eyes and the adult antennae are completely new structures in the adult insect. A neural system is, however, metabolically very costly to maintain [12] and will therefore be subject to conflicting selective pressures, on the one hand to save energy and on the other hand to optimize the need for reliable information processing. Therefore, it can be hypothesized that in pupal diapausers, overall development of the brain will be arrested during diapause and only structures directly associated with maintenance of diapause metabolism or needed for the termination of diapause will be developed at an early diapause stage. By contrast, structures only needed in the adult insect will develop as part of ontogenetic development after diapause.

The green veined white, *Pieris napi* Linnaeus (Lepidoptera: Pieridae), is a butterfly with facultative diapause that shows variation in generation number across Europe. Diapause is initiated in larvae in response to shortening day length [13,14]. Whereas pupal development without diapause generally lasts about two weeks, pupal diapause may last two to six months [15]. Thus, the lifespan of individuals can vary from about 1.5 months in a directly developing individual to over 10 months in an individual undergoing diapause [5].

In this study, we performed immunohistochemical stainings with subsequent confocal sectioning and reconstructions and made volumetric comparisons of a number of identifiable brain structures among *P. napi* individuals undergoing direct or diapause development in a time-series experiment. We tested (i) if and (ii) when brain development is arrested in diapausing pupae and (iii) if the sequential development of adult-like brain structures is similar in the two developmental pathways.

## 2. Material and methods

### (a) Animal rearing

Eggs of *P. napi* were collected in 2013 and 2014 from wild plants in two sites (approx. 20 km apart) in Skåne, southern Sweden (Kullaberg; 56°18′ N, 12°27′ E and Vejbystrand; 56°18′ N, 12°46′ E) and brought to the Department of Zoology at Stockholm University. These were left to develop on *Armoracia rusticana* (Brassicales: Brassicaceae) at a long day photoperiod (22 L : 2 D, 20°C) in a mixed group. Adult butterflies of both sexes were kept in cages (0.8 × 0.8 × 0.5 m) and provided with *A. rusticana* for oviposition at 25°C under long day conditions (under 400 W metal halide lamps) and fed sugar water. Their offspring were mass-reared to adulthood on *A. rusticana* at a long day photoperiod (as above) and re-mated under similar conditions to their parents. For the experiments, their offspring (i.e. the F_2_ generation) were reared to pupation in groups of five on *A. rusticana* leaves under conditions inducing either diapause (10 L : 14 D, 20°C) or direct development (22 L : 2 D, 20°C) in climate cabinets (KB8400 L, Termaks, Bergen, Norway). Diapausing individuals were kept for 10 days at diapause-inducing conditions, then moved for 7 days to dark 10°C cabinets and finally to dark 2°C cabinets. After 144 days, they were again moved to 10°C cabinets for 7 days and then to 22 L : 2 D, 20°C, cabinets until eclosion. Adults in both direct and diapause pathways were kept at long day conditions (as above) and fed only water.

### (b) Sampled stages

Sampling was designed to cover metamorphosis and diapause as well as important transitions within both pathways. For both pathways, we investigated the brain structures 0, 3, 6 and 9 days after pupation (P0, P3, P6 and P9 henceforth). Additionally, we investigated newly eclosed (2-day-old) adults from both pathways and 69- and 114-day-old pupae in diapause (P69 and P114 henceforth). Based on previous data, we know diapause is terminated in the studied population after approximately 114 days [5,15]. Therefore, we took out post-termination pupae from the cold after 144 days and sampled after 4 days at 10°C (P148 henceforth), 7 days at 10°C plus 1 day at 20°C (P152 henceforth) and finally, 7 days at 10°C plus 7 days at 20°C (P158 henceforth). The general idea, based on previous research [15], was to sample comparable developmental stages in both the direct (P3, P6 and P9) and diapause (P148, P152 and P158) pathways.

### (c) Immunohistochemical stainings of the brain

Heads or the anterior body of the different stages of butterflies were fixed overnight at 4°C in 4% paraformaldehyde in 0.1 M sodium phosphate buffer (PB). After careful rinsing in PB, the brains were dissected in phosphate-buffered saline with 0.25% Triton-X (PBS-Tx). Thereafter, the brains were preincubated overnight in 5% normal goat serum in PBS-Tx. Then brain tissue was incubated for 72 h in mouse monoclonal anti-synapsin (anti- SYNORF1, 1 : 20; Developmental Studies Hybridoma Bank, Iowa City, IA, USA). After rinsing in PBS-Tx, we used an Alexa 546-tagged secondary antibody (1 : 1000; Invitrogen) for detection of antiserum. Finally, the brains were rinsed, dehydrated in an ascending series of ethanol concentrations and cleared and mounted in methyl salicylate. For some of the stages, we also used a rabbit polyclonal antibody against the neuropeptide allatostatin-A (AST-A, 1 : 1000, Agricola) and an Alexa 488-tagged secondary antibody (1 : 1000; Invitrogen) for detection.

### (d) Scanning and reconstruction

**Figure 1.**
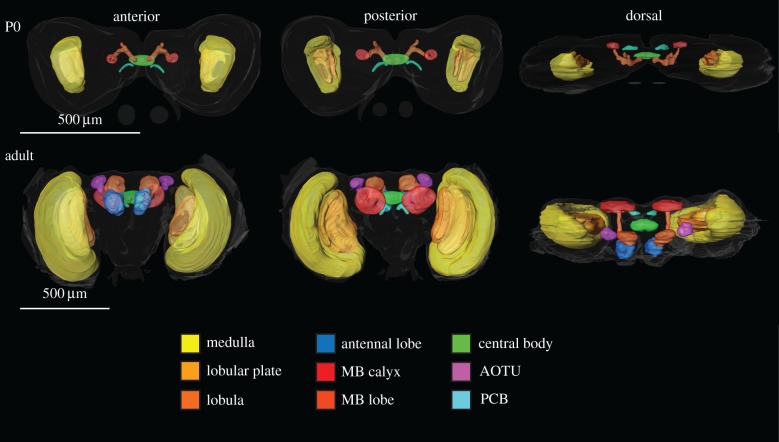
Surface reconstructions of brain structures underlying subsequent volumetric measurements. The upper panel shows a reconstruction of the brain from a diapausing pupa at stage P0 from three different directions. The lower panel shows a reconstruction of an adult brain viewed from the same three directions. Outlines of the entire brain are shown as well as nine different brain structures, which are colour-coded according to the legend below. Note that the antennal lobes and the AOTUs are lacking in the P0 brain. MB, mushroom body; AOTU, anterior optic tubercle; PCB, protocerebral bridge. Note the different scales for the two stages.

The brains were scanned with a Zeiss LSM 780 META confocal laser scanning microscope (Zeiss, Jena, Germany) and images were obtained with a 10× air objective. The resolution of all images was 1024 × 1024 pixels. Scanned stacks (−100 sections, step size 3 µm) were then imported into Amira 3D analysis software v. 6.0 (FEI, Oregon, USA), and we used the segmentation tool to manually label all identifiable structures. The volumes of the structures were then calculated using the measurement tool. Five brains from each timepoint and pathway (i.e. 75 individuals) were reconstructed and volumetrically analysed. All measurements reflect the summed volume of the respective structure from both hemispheres, except the two unpaired structures, the central complex and the protocerebral bridge. In some individuals, only one of the paired structures was measured (e.g. due to damage) and thus multiplied by two. In addition to the entire brain size, we measured the volume of the medulla, lobular plate, lobula, anterior optic tubercle, mushroom body calyx, mushroom body lobe system including the peduncle, antennal lobe, central body and the protocerebral bridge (figure 1).

### (e) Statistics

The data were analysed with a series of generalized linear models where brain region was added as dependent variable and age-group (0, 3, 6, 9, 69, 114, 148, 152, 158 and adult) as well as pathway (direct, diapause) were added as factorial explanatory variables. Prior to running these tests, the data were log-transformed due to large difference between the immature and adult brain sizes. As the age-group×pathway interaction was significant for all brain regions (electronic supplementary material, table S1), post hoc tests employing the Bonferroni corrections for multiple-group comparisons were performed to test differences among the following key groups. (i) To test when diapause and direct brains diverge, we compared P0–P9 direct versus diapause. (ii) To test how much the brain developed early in diapause, we compared P0 versus P3. (iii) To then test when the brain is arrested in development, we compared P3–P114. We specifically looked for the final age when no change in size is seen at later timepoints. (iv) To see if brain development is similar between the post-diapause and direct pupae, we compared P9 in direct pupae to P158 in diapause pupae. (v) Finally, to test whether adults differed depending on developmental pathway, we compared diapause and direct adult brains. All statistical tests were performed in SPSS v. 24.0 (IBM Corp., Armonk, NY, USA).

## 3. Results

### (a) Summary of results

The goals of this study were to assess (i) if and (ii) when brain development is arrested in diapausing pupae and (iii) the state of development of the different quantifiable brain regions and substructures during diapause.

**Figure 2.**
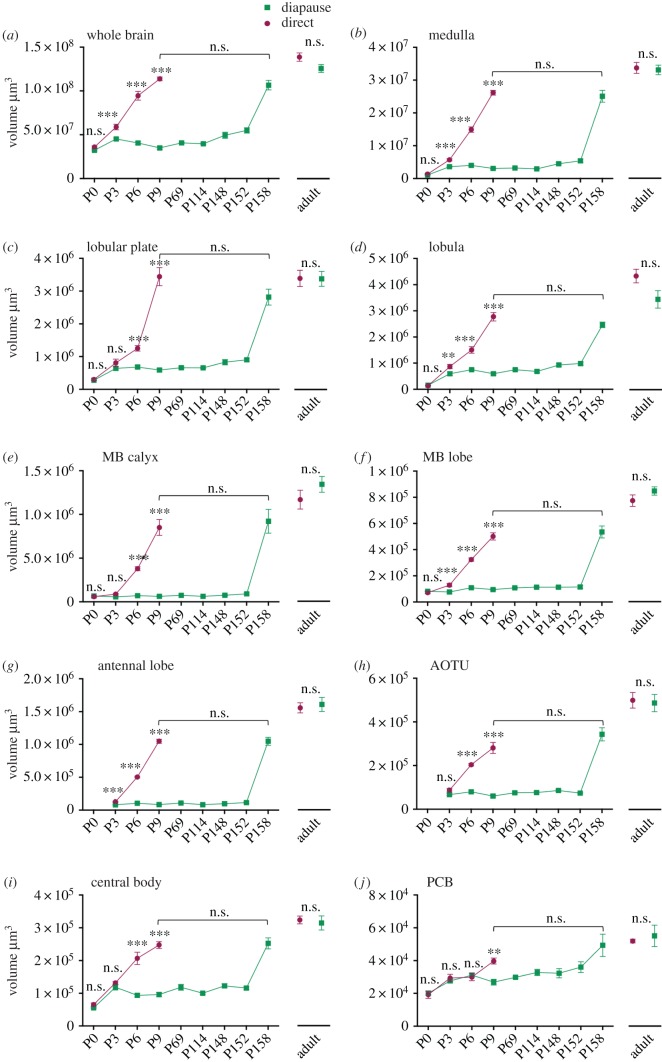
Development of the whole brain volume and the volumes of the reconstructed brain structures in directly developing and diapausing butterflies. To the right of each graph, the sizes of brain structures in adult butterflies are shown. Note that uncorrected volumes are depicted in figures, even though log-transformed data were used for analyses. Data depicted as mean ± s.e.m. ***p < 0.001; **p < 0.01; n.s., not significant. MB, mushroom body; AOTU, anterior optic tubercle; PCB, protocerebral bridge. (Online version in colour.)

Overall brain development was parallel in directly and diapausing pupae until P3, when development was arrested in diapausing pupae (figure 2*a*). For individual brain regions there was more variation, but generally growth was arrested at P3 (see individual brain region descriptions below). After development was arrested, no growth was observed in the individual brain regions until after diapausing pupae were removed from the cold (figure 2*b–h*), when development quickly resumed a similar trajectory to that seen in direct developing pupae. Indeed, at P158, no difference in size could be detected any longer in the whole brain nor any individual brain region compared with P9 direct developing pupae (figure 2). Not surprisingly, adult brains of the two pathways did not differ in whole brain volume (figure 2*a*) nor any of the analysed substructures (figure 2*b–j*) and adults will therefore not be discussed further in this section.

**Figure 3.**
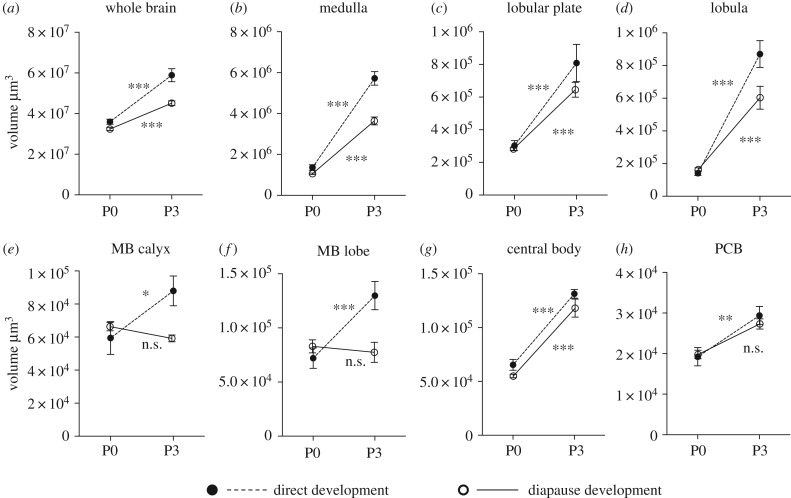
Development of brain structures between P0 and P3 in diapausing and direct developing pupae. Note that uncorrected volumes are depicted in figures, even though log-transformed data were used for analyses. Data depicted as mean ± s.e.m. *p < 0.05, **p < 0.01, ***p < 0.001, n.s., not significant. MB, mushroom body; PCB, protocerebral bridge.

**Figure 4.**
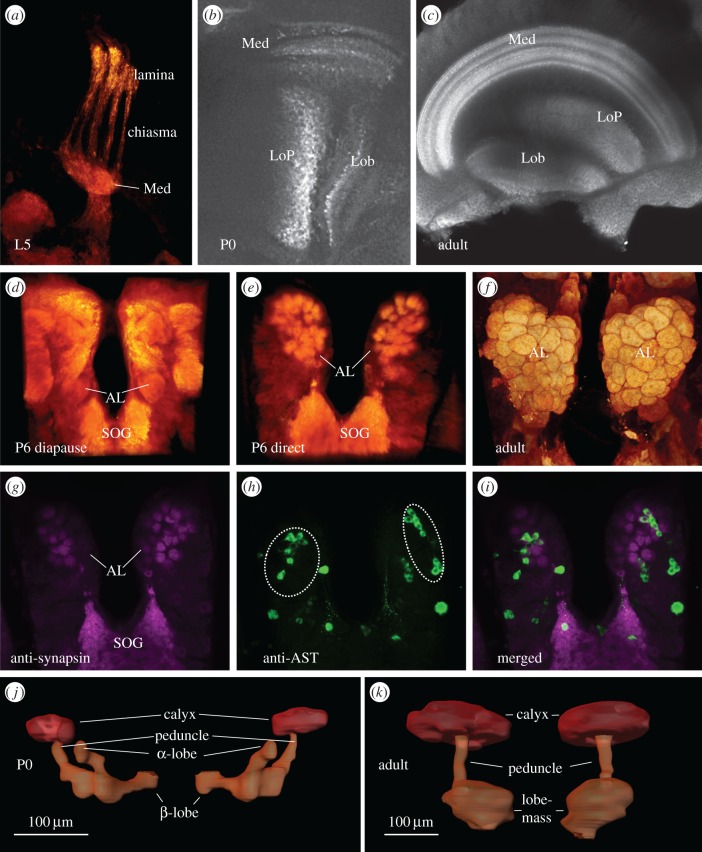
Development of structures related to sensory processing. (*a–c*) The development of the optic lobe shown as single sections from stacks of confocal images. The larval optic lobe (*a*) is still visible during P0 but not at later stages. Already by P0 (*b*), the adult-like optic lobe has developed with all substructures including a striated medulla (note that the larval optic lobe is not visible at this focal plane). (*d–i*) Development of the antennal lobes (AL). The anatomy of the AL differs dramatically at P6 (*d*,*e*) between diapausing and directly developing butterflies. At P6, glomeruli are clearly visible in directly developing brains but not in diapausing ones. (*f*) The ALs in an adult butterfly with the typical glomerular substructures. (*d–f*) Volume-rendered images from confocal stacks. (*g–i*) Double immunostainings with anti-synapsin and anti-AST-A are shown. Note the approximately 10 cells in the lateral cell cluster innervating the AL (depicted by a dotted white circles). These cells are not detectable in diapausing brains until P158. (*j–k*) Development of the mushroom bodies (MB). In P0, the MBs consist of two distinguishable lobes (α and β), which resemble the larval MBs. At P3, the two lobes fuses into a common adult-like lobe (lobe mass), as in (*k*). Med, medulla; L5, larval stage 5; LoP, lobular plate; Lob, lobula; SOG, suboesophageal ganglion; AL, antennal lobe; AST, allostatin. (Online version in colour.)

Comparing development of brain regions early in pupation (P0 versus P3) between the pathways revealed differences in brain regions related to different sensory functions. Regions participating in visual information processing (medulla, lobular plate, lobula) grew and differentiated significantly in both pathways (figures 2–4). However, regions related to olfaction (antennal lobe, mushroom body calyx and lobe) only grew or differentiated in direct developing pupae from P0 onwards and remained in developmental arrest in diapausing pupae until after diapause termination (figures 2–4).

### (b) Description of growth in individual brain structures

#### (i) Optic lobes

The larval optic lobes anatomy showed a finger-like structure [16] (figure 4*a*). These larval optic lobes were still faintly visible in some specimens during P0 in conjunction with the newly developed adult-like optic lobes. However, from P3 onwards, the larval optic lobes could no longer be distinguished. Already at P0, an adult-like optic lobe structure was visible and responsible for most of the brain expansion from the larval brain in both pathways. All four optic lobe subunits could be observed. However, as it often was difficult to determine the exact outlines of the lamina, we decided not to include this subunit in the volumetric analyses. The other three discernible subunits of the optic lobes (medulla, lobular plate and lobula), however, were analysed. These subunits were all clearly visible at P0 in both pathways. In addition, the typical layered structure of the medulla had developed at this early stage (figure 4*b*). In diapausing pupae, the three optic subunits grew between P0 and P3 (figure 3*b–d*) and then no further growth was observed until P158.

#### (ii) Mushroom bodies

The mushroom bodies in *P. napi* consist of a calyx, a peduncle and a lobe system. At P0, the lobe system consisted of two clearly discernible lobes, α- and β-lobe [17] (figure 4*j*) identical to those seen during all larval stages (M.A.C. 2016, personal observation). However, between P0 and P3, the lobes fuse to one larger lobe mass (figure 4*k*). Moreover, the calyx moves from a dorsal to a more posterior position (not shown). From P0, we measured the volume of the calyx and the summed volume of the peduncle and lobe system. In diapausing pupae, none of the mushroom body substructures grew between P0 and P3 (figure 3*e–f*), and not until after diapause termination could any growth be observed.

#### (iii) Antennal lobes

The antennal lobes could not be observed at P0 but were clearly identifiable at P3 in both pathways, but no glomerular subdivisions could be seen at this stage. However, from P6 and onwards, glomeruli were clearly discernible in directly developing pupae (figure 4*e*). The number of glomeruli, about 65, was similar to the amount found in the adult antennal lobe [18] (figure 4*f*), though the exact number was difficult to count due to diffuse membrane borders, particularly at the posterior part of the lobe. In diapausing pupae, the non-glomerular antennal lobe (figure 4*d*) persisted until after termination of diapause (P158).

In addition, we stained a number of brains with an antibody against allatostatin-A, which is expressed in local interneurons in the antennal lobe of adult insects, including butterflies [19,20]. From P6 onwards, about 10 somata of local interneurons were immunoreactive in a lateral cell cluster of the directly developing brains (figure 4*g–i*). These local interneurons persisted throughout development. By contrast, in diapausing pupae, we could not observe any AST-A immunostaining in the antennal lobes until after diapause termination (P158). AST-A staining was, however, observed in other brain neuropils.

#### (iv) Anterior optic tubercle

The anterior optic tubercle was not clearly visible at P0 (nor in larvae; M.A.C. 2016, personal observation), thus the volume of this structure was only measured from P3 onwards. This structure is actually a complex consisting of one large and three smaller glomeruli, as clearly seen in adult brains. At earlier stages, however, the borders were often too diffuse to be able to identify the substructures. Therefore, we only measured the volume of the entire structure. This neuropil remained locked in developmental arrest in diapausing pupae at P6.

#### (v) Central body

This unpaired medially located neuropil consists of several sublayers, which were clearly distinguishable in the brains of adults. However, as the borders between sublayers were relatively diffuse and not easily distinguishable at earlier stages, we only analysed the volume of the entire structure. The central body grows between P0 and P3 in diapausing brains (figure 3*g*), did not differ in size from directly developing brains at P0 and P3 (figure 2*i*) and finally became arrested in development at P6 in diapause. The central body was also observed at all larval stages (M.A.C. 2016, personal observation).

#### (vi) Protocerebral bridge

Finally, the protocerebral bridge is an unpaired structure located on the posterior side of the brain. The protocerebral bridge was observed in all stages from the newly emerged larva onwards (not shown). This neuropil did not grow significantly from P0 to P3 in diapausing pupae (figure 3*h*) and there was no significant difference between the pathways until P9 (figure 2*j*), when development was arrested in diapausing pupae.

## 4. Discussion

Here, we show for the first time a detailed study of pupal brain development during diapause. As predicted, the development of the brain was arrested at an early stage in diapausing pupae. The brains of both directly developing and diapausing pupae grew in size up to stage P3 and all adult structures we analysed were clearly discernible at that stage. However, while the size of the diapausing brain remained more or less unaltered from P3 until after diapause termination, the directly developing brain increased continuously in size until eclosion.

During the first 3 days after pupation, the entire brain went through a dramatic morphological change when compared with the larval stage. First, the adult-like optic lobes were already developed at P0. The larval finger-like optic lobe was still visible at P0 but simultaneously adult optic lobes became apparent, which developed from the optic anlagen [21]. At P3, the larval optic lobes were no longer visible. Second, the suboesophageal ganglion, the most anterior of the larval ventral ganglia, which was clearly separated from the brain in larvae, started moving towards the brain and was at P3 completely fused. The fusion of the brain and the suboesophageal ganglion is mainly responsible for the volumetric growth of the total brain between P0 and P3. Third, the mushroom bodies changed their morphology in that the larvae have two clearly separated lobes (α and β) that during the first 3 days of pupation fuse into a larger lobe mass. Fourth, antennal lobes could not be distinguished at P0 but were clearly visible at P3. And last, the anterior optic tubercle, a structure not present in larvae, becomes apparent at P3.

Interestingly, development of the diapausing brain completely stops already at an early stage, as expected [12], but only after most of the adult brain structures become apparent. The visual system of diapausing pupae goes through a dramatic morphological change already at P0, when a miniature adult optic lobe was developed with the four subdivisions clearly discernible. Moreover, the adult-like layered structure of the medulla was clearly visible at this stage. A layered medulla is supposed to be required for processing of visual information [22]. Different layers are devoted to the processing of colour or motion information and interneurons confined to the medulla promote processing within and between layers. Another structure associated with visual processing in adult insects, the anterior optic tubercle, was visible at P3 in both pathways. The anterior optic tubercle has recently been shown to be involved in processing of chromatic information with a spatial representation of wavelength information in the four individual glomeruli of this neuropil [23]. Together, these results mean that a diapausing pupa of *P. napi* could potentially process chromatic information.

The indication that the visual system may be functional throughout diapause is important because many insects are sensitive to light during diapause and indeed use photoperiod to promote development in spring [24]. During diapause induction, photoperiodic input has been shown to be processed through the light-sensitive protein cryptochrome in several insect species [25–27]. Cryptochrome is a blue-light photopigment [28] and thus the anterior optic tubercle neuropils described above could be involved in counting daily photoperiodic cycles. However, pilot experiments do not suggest any strong photoperiodic sensitivity during diapause in *P. napi* (D. Posledovich 2017, unpublished data). Even though photoperiod itself might not be the cue for diapause termination, light might still be important for the entrainment of the circadian clock and thus counting light–dark cycles, i.e. days in diapause [9]. As pupae can diapause in locations hidden from light (i.e. under snow, in leaf litter), a developed visual system could also indicate the presence of a functional circadian clock neural circuitry that could count days according to an endogenous rhythm without need for visual input. It would in either case be important to further study the localization and development of circadian neurons [8] during diapause development.

In neither pathway could we detect any antennal lobes at P0, whereas these were visible during all larval stages. This could imply that the antennal lobes are structures that do not survive metamorphosis, which would be in contrast to studies in the moth *Manduca sexta*, where it has been demonstrated that most larval neurons that innervate the larval antennal lobe indeed persist through metamorphosis and together with newborn neurons build the adult antennal lobe [29–32]. However, in accordance with our study, synapses in the antennal lobe of *M. sexta* were not visible until P4 [33]. This means that the antennal lobe may still be present in *P. napi* but could not be detected due to weak anti-synapsin staining. In the directly developing brain, glomerular formations in the antennal lobe were seen at P6 but not at P3. The glomerular borders were, however, not as clearly distinguishable as in the adult antennal lobe. The size of the antennal lobe and its glomeruli increased from P6 to the adult. By contrast, during diapause, glomeruli do not develop until between P152 and P158. As predicted, the adult antennal lobe does not differ between the pathways, suggesting strong canalization of the adult phenotype [34].

Whereas the development of the visual system suggests a functional role for this sensory modality even in the diapausing pupa, the olfactory system seems to remain undeveloped and thus likely non-functional until after termination of diapause. We base this conclusion on the fact that neither glomerular structures nor AST-A immunoreactive local interneurons, which are needed for olfactory information processing, could be observed until P158. This is not surprising as volatile molecules can hardly enter the pupal cuticle and be transmitted to a receptor site and it would thus be an energetic waste with a fully developed olfactory system.

Glomeruli are only formed after axons from the developing antennae start innervating the antennal lobe. In *M. sexta*, in which the pupal stage lasts for about 18 days, axonal innervation takes place at about P5 and immature protoglomeruli are first observed at P6 [35]. We can thus assume that axonal ingrowth starts sometime between P3 and P6 in directly developing pupae. By contrast, in the diapausing pupae, no glomerular structures are observed until P158 and we can therefore assume that axonal innervation takes place between P152 and P158.

The glomerular formation in the antennal lobe further coincided with the expansion of the mushroom bodies. In contrast with visually related neuropils, the mushroom bodies did not grow between P0 and P3 in diapausing pupae (figure 3*e–f*). This is not surprising because most of the input to the calyx of the mushroom body stems from projection neurons of the antennal lobe [36]. Projection neurons have been found to take part in the formation of glomeruli in the antennal lobe of *M. sexta* [37]. As the expansion of the mushroom bodies coincided with glomerular formation at P6 in the directly developing *P. napi*, it is not unlikely that a projection neuron connection has been established between the neuropils and is responsible for synaptic arbourization in the mushroom body calyx, which will cause the growth.

The mushroom bodies participate in memory formation, particularly in olfactory oriented learning [38]. Furthermore, a few studies have suggested that information acquired during larval stages can be retrieved in adults, i.e. a transmetamorphic memory [39–41]. In our study, we found a continuous development of the mushroom bodies from larval stages through P0 to adults, which suggests that memories that have been established during larval feeding may survive to the adult butterflies and could potentially affect female oviposition choice. If so, an obvious question is whether such memories would differ between directly developing and diapausing pupae, as the mushroom body development in the latter pathway remains arrested during several months.

## 5. Conclusion

Our results show that brain development is arrested at around P3 in diapausing pupae. No growth or differentiation at all is observed during diapause, but only after temperatures increase, far later than diapause termination. We also show a pronounced difference between direct and diapausing pupae in the development of sensory structures related to vision and olfaction. While sensory structures related to vision develop similarly in both pathways up to P3, no development of structures related to olfaction is observed in diapausing pupae. It is of course premature to assign functional roles of sensory processing based on correlational morphological analyses, but this study suggests that a relatively well-developed visual system might be important for normal diapause development. As the mechanisms behind diapause development and time-keeping during endogenous diapause are poorly understood, this study opens an interesting avenue of research to pursue.

## Supplementary Material

Supplement table S1

## Data accessibility

All relevant data associated with this study are available from the Dryad digital repository: http://dx.doi.org/10.5061/dryad.f36mq [42].
