# Supplementary material for: Idiosyncratic development of sensory structures in brains of diapausing butterfly pupae: implications for information processing
Source: Proc Biol Sci. 2017 Jul 5;284(1858):20170897. doi: 10.1098/rspb.2017.0897 (PMC5524504; doi:10.1098/rspb.2017.0897)
Supplement: Supplement table S1 [file rspb20170897supp1.docx]

Supplementary information for the article:

**Idiosyncratic development of sensory structures in brains of diapausing butterfly pupae: implications for information processing**

Philipp Lehmann, Sören Nylin, Karl Gotthard, Mikael A Carlsson

Department of Zoology, Stockholm University, Stockholm, Sweden

Corresponding author: Mikael A Carlsson, email: [mikael.carlsson@zoologi.su.se](mailto:mikael.carlsson@zoologi.su.se)

**Extended statistical analysis**

This supplementary table contains the results of a generalised linear model (GLM) where brain region was added as dependent variable and age-group (0, 3, 6, 9, 69, 114, 148, 152, 158 and adult) as well as pathway (direct, diapause) were added as factorial explanatory variables. Prior to running these tests the data were log-transformed due to large difference between the immature and adult brain sizes. As can be seen from Table S1, the age-group*pathway interaction was significant for all brain regions. Therefore post-hoc tests employing Bonferroni corrections for multiple-group comparisons were performed to test differences among key groups, as described in the statistics section in the article. The results of these tests can be seen in Figs. 2 & 3.

**Table S1** Generalised linear models (GLM) testing the effect of pathway and time on brain development in *Pieris napi* undergoing direct or diapause development.

| Structure | Effect | Wald χ2 | DF | P |
| --- | --- | --- | --- | --- |
| WB | Intercept | 2875734,076 | 1 | <0.001 |
|  | Age | 1813,423 | 9 | <0.001 |
|  | Pathway | 429,721 | 1 | <0.001 |
|  | Age*Pathway | 331,620 | 4 | <0.001 |
| MED | Intercept | 1081684,978 | 1 | <0.001 |
|  | Age | 5036,277 | 9 | <0.001 |
|  | Pathway | 602,973 | 1 | <0.001 |
|  | Age*Pathway | 530,560 | 4 | <0.001 |
| LOP | Intercept | 436700,159 | 1 | <0.001 |
|  | Age | 1475,726 | 9 | <0.001 |
|  | Pathway | 122,939 | 1 | <0.001 |
|  | Age*Pathway | 186,620 | 4 | <0.001 |
| LOB | Intercept | 570432,127 | 1 | <0.001 |
|  | Age | 2936,537 | 9 | <0.001 |
|  | Pathway | 172,928 | 1 | <0.001 |
|  | Age*Pathway | 180,310 | 4 | <0.001 |
| AOTU | Intercept | 292811,682 | 1 | <0.001 |
|  | Age | 1076,513 | 8 | <0.001 |
|  | Pathway | 157,968 | 1 | <0.001 |
|  | Age*Pathway | 105,913 | 3 | <0.001 |
| MBCX | Intercept | 283257,489 | 1 | <0.001 |
|  | Age | 2477,004 | 9 | <0.001 |
|  | Pathway | 278,459 | 1 | <0.001 |
|  | Age*Pathway | 450,540 | 4 | <0.001 |
| MBL | Intercept | 389613,503 | 1 | <0.001 |
|  | Age | 1782,668 | 9 | <0.001 |
|  | Pathway | 189,235 | 1 | <0.001 |
|  | Age*Pathway | 248,820 | 4 | <0.001 |
| AL | Intercept | 635810,751 | 1 | <0.001 |
|  | Age | 4686,590 | 8 | <0.001 |
|  | Pathway | 945,988 | 1 | <0.001 |
|  | Age*Pathway | 728,929 | 3 | <0.001 |
| CB | Intercept | 658328,536 | 1 | <0.001 |
|  | Age | 1200,624 | 9 | <0.001 |
|  | Pathway | 153,061 | 1 | <0.001 |
|  | Age*Pathway | 135,100 | 4 | <0.001 |
| PCB | Intercept | 292147,077 | 1 | <0.001 |
|  | Age | 258,377 | 9 | <0.001 |
|  | Pathway | 2,006 | 1 | 0.157 |
|  | Age*Pathway | 15,703 | 4 | 0.003 |

WB = whole brain, MED = medulla, LOP = lobular plate, LOB = lobula, AOTU = anterior optical tubercle, MBCX = mushroom body calyx, MBL = mushroom body lobe, AL = antennal lobe, CB = central body, PCB = protocerebral bridge
